# Impact of age on the cumulative risk of transformation in patients with chronic myelomonocytic leukaemia

**DOI:** 10.1111/ejh.13647

**Published:** 2021-06-01

**Authors:** Sigrid Machherndl‐Spandl, Eva Jäger, Agnes Barna, Michael Gurbisz, Renate Marschon, Temeida Graf, Elmir Graf, Christoph Geissler, Gregor Hoermann, Thomas Nösslinger, Michael Pfeilstöcker, Peter Bettelheim, Otto Zach, Ansgar Weltermann, Sonja Heibl, Josef Thaler, Armin Zebisch, Heinz Sill, Reinhard Stauder, Gerald Webersinke, Rajko Kusec, Ernst Ulsperger, Bruno Schneeweiss, Leopold Öhler, Ulrich Germing, Peter Valent, Heinz Tüchler, Klaus Geissler

**Affiliations:** ^1^ Department of Internal Medicine I with Hematology Stem Cell Transplantation Hemostasis and Medical Oncology Ordensklinikum Elisabethinen Hospital Linz Austria; ^2^ Department of Laboratory Medicine Medical University of Vienna Vienna Austria; ^3^ Blood Transfusion Service Blood Transfusion Service for Upper Austria Austrian Red Cross Linz Austria; ^4^ Laboratory for molecular and genetic diagnostics Ordensklinikum Linz Linz Austria; ^5^ Department of Internal Medicine V with Hematology, Oncology and Palliative Medicine Hospital Hietzing Vienna Austria; ^6^ Department of Laboratory Medicine Hospital Hietzing Vienna Austria; ^7^ MLL Munich Leukemia Laboratory Munich Germany; ^8^ Ludwig Boltzmann Institute for Hematology and Oncology (LBI HO) Medical University of Vienna Vienna Austria; ^9^ Department of Internal Medicine III Hanusch Hospital Vienna Austria; ^10^ Department of Internal Medicine IV Hospital Wels‐Grieskirchen Wels Austria; ^11^ Division of Hematology Medical University of Graz Graz Austria; ^12^ Otto‐Loewi Research Centre for Vascular Biology, Immunology and Inflammation Division of Pharmacology Medical University of Graz Graz Austria; ^13^ Internal Medicine V with Hematology and Oncology Medical University of Innsbruck Innsbruck Austria; ^14^ School of Medicine University Hospital Dubrava University of Zagreb Zagreb Croatia; ^15^ Department of Internal Medicine Hospital Horn Horn Austria; ^16^ Department of Internal Medicine Hospital Kirchdorf Kirchdorf Austria; ^17^ Department of Internal Medicine/Oncology St. Josef Hospital Vienna Austria; ^18^ Department of Hematology, Oncology, and Clinical Immunology Heinrich‐Heine‐University Düsseldorf Germany; ^19^ Division of Hematology and Hemostaseology Department of Internal Medicine I Medical University of Vienna Vienna Austria; ^20^ Sigmund Freud University Vienna Austria

**Keywords:** age, blast cell count, chronic myelomonocytic leukaemia, cytogenetics, mutations, score

## Abstract

In older patients with chronic myelomonocytic leukaemia (CMML) and limited life expectancy due to age and or comorbidities, it is particularly important to consider the risk of transformation for individualised treatment decisions. There is limited information on potential differences between younger and older CMML patients regarding the cumulative risk of transformation as well as haematological, molecular and biologic characteristics. We analysed data from the Austrian Biodatabase for CMML (ABCMML) to compare these parameters in 518 CMML patients. Categorisation of patients into 3 age‐related groups: <60 years, 60‐79 years and ≥80 years, showed a significantly lower risk of transformation at higher age by competing risk analysis, with a 4‐year risk of 39%, 23% and 13%, respectively (*P* < .0001). The lower probability of transformation was associated with a lower percentage of blast cells in the peripheral blood (PB) of older patients. Furthermore, we provide a simple score based on age, PB blasts and platelet counts that allowed us to define subgroups of CMML patients with a different cumulative transformation risk, including a low‐risk group with a transformation risk of only 5%. Our findings may facilitate reasonable treatment decisions in elderly patients with CMML.

## 1 INTRODUCTION

Chronic myelomonocytic leukaemia (CMML) is a hematopoietic malignancy with a male predominance and a median age at diagnosis of 70 ranging from 16 to 93 years.^1^ This clonal disease is characterised by overlapping features of myelodysplastic syndromes (MDS) and myeloproliferative neoplasms (MPN), and an inherent risk of transformation to secondary acute myeloid leukaemia (sAML).^2^, ^3^, ^4^, ^5^, ^6^, ^7^ The median overall survival of CMML patients is about 30 months. Allogeneic hematopoietic stem cell transplantation, which is the only curative therapy, is rarely feasible because of age and/or comorbidities. In patients ineligible for stem cell transplantation, intensive chemotherapy results in low response rates and short response duration.^8^ Hydroxyurea is often used to control myeloproliferation in CMML.^9^ The cytidine analogues azacytidine (AZA) and decitabine (5‐aza‐2'‐deoxycytidine) have demonstrated some efficacy in delaying disease course in advanced CMML and were approved for the treatment of CMML.^10^, ^11^, ^12^

In the “Austrian Biodatabase for Chronic Myelomonocytic Leukemia” (ABCMML), we retrospectively and prospectively collect haematological, clinical, molecular and biologic information of patients with CMML from different centres in a real‐life setting to elaborate epidemiologic, prognostic and predictive information for specific subgroups and to get insights into the pathophysiology of the disease. We have recently demonstrated that regarding patient characteristics and established prognostic parameters our patient cohort is consistent with other published CMML patient series and therefore may be an adequate data source for further research.^13^

In CMML patients with limited life expectancy due to old age and or comorbidities, it is particularly important to consider the aggressiveness of the disease and the risk of transformation for individualised treatment decisions. There is limited information on potential differences between younger and older CMML patients regarding risk of transformation as well as haematological, molecular and biologic characteristics. In this study, therefore, we analysed data from the ABCMML to compare these parameters in 518 CMML patients categorised into sub‐groups according to different age.

## 2 PATIENTS AND METHODS

The ABCMML was established to retrospectively collect real‐life data, including epidemiologic, haematological, biochemical, immunophenotypic, cytogenetic, molecular and biologic parameters in 606 patients with CMML from 14 different centres seen between 1988 and 2018. Details of the biodatabase regarding patient characteristics and sample numbers, data sources and methods have been published recently.^13^ Clinical and laboratory parameters were obtained at time of referral to centres that, in most instances, coincided with the time of bone marrow biopsies. Data were checked for accuracy and consistency. Data curation included the extraction of discrete data elements from patient records, a check for accuracy and consistency of data and a verification that baseline data were reflective of CMML that was strictly defined according to WHO criteria.^6^ Patients with a history of antecedent CMML and 20% or more blasts in peripheral blood (PB) and/or bone marrow (BM) were uniformly considered as CMML‐derived AML. PB and/or BM samples were taken after written informed consent was provided by patients. Internal review board approval was obtained at each institution.

### 2.1 Cytogenetic analyses

Cytogenetic analysis was performed using G‐banding according to standard techniques on BM cells 24 to 48 hours in unstimulated culture. Chromosome aberrations were classified according to the International System for Human Cytogenetic Nomenclature (ISCN). CMML‐specific cytogenetic risk classification was low for normal karyotype and isolated ‐Y, intermediate for other abnormalities and high for trisomy 8, complex karyotype (≥3 abnormalities) and abnormalities of chromosome 7.^14^

### 2.2 Semisolid in vitro cultures

Colony‐forming unit‐granulocyte‐macrophage (CFU‐GM) growth was assessed in semisolid cultures without growth factors as previously described in one central laboratory.^15^ Cultures were plated in duplicates or triplicates, respectively, at 25‐100 × 10^3^ PB mononuclear cells (MNC) per millilitre. In some cases, the numbers of PB MNC chosen in our experiments were based on the colony growth in prior cell cultures in the respective patient in order to optimise evaluation of CFU‐GM formation. Plates were incubated at 37°C, 5% CO2, and full humidity. After a culture period of 14 days, cultures were examined under an inverted microscope. Aggregates with more than 40 translucent, dispersed cells were counted as CFU‐GM. CFU‐GM data are expressed as mean values from cultures.

### 2.3 Molecular analysis

Molecular analyses were performed using next‐generation sequencing (NGS) with amplicon‐based target enrichment. Genomic DNA was isolated from MNC fractions of PB or BM samples according to standard procedures. The gene panels, databases and software tools for variant annotation and interpretation used have been reported elsewhere.^13^ Only pathogenic variants were taken and a VAF of 5% or higher was considered as positive for analysis.^16^

### 2.4 Statistical analysis

For description of categorical features, counts and percentages are given, and medians plus ranges for continuous variables. As a measure of correlation, Kendall's tau was used. Tests for differences by age are based on Kendall's tau.

The relation of age with the actual risk of transformation was described by penalised splines (psplines) without as well as with stratification by the Mayo risk score, WBC, cytogenetic risk and *ASXL1*.

The risk of transformation was analysed in a competing risk framework,^17^ regarding death without transformation as competing event.

Differences were tested according to Gray.^18^ The development of the proposed score was internally validated by bootstrapping each step of the construction.

Two‐sided *P*‐values < .05 were considered significant. Confidence limits refer to a 95% level. In line with the essentially exploratory nature of the study, no adjustment for multiple testing was applied.

All analyses were conducted with the statistics software R 3.6.2, including the packages "survival" and "cmprsk".^19^, ^20^, ^21^

## 3 RESULTS

Adequate data were obtained from 518 out of the 606 registered patients with CMML. The patient characteristics of the total cohort have been described previously.^13^

Since the impact of age on the cumulative risk of transformation was a main focus of this study, we first looked for potential differences in CMML‐related features between older and younger CMML patients. In Tables 1 and 2, comparisons of laboratory and molecular characteristics in patients with CMML categorised into age younger than 60 years vs age 60‐79 vs 80 years or older are shown. Older patients ≥60 years had a lower number of peripheral blood blasts as compared to younger patients (see also Table S1), whereas all other parameters were neither substantially nor significantly different between the age categories.

**TABLE 1:** Comparison of epidemiologic, laboratory, biologic and molecular characteristics in patients with CMML stratified by age younger than 60 years vs 60‐79 years vs 80 years or older

Variables	All patients N = 518	Patients <60 years N = 57	Patients 60‐79 years N = 357	Patients ≥80 years N = 104	*P* Value
Median OS (months)	27.6	21.1	29.4	27.0	
Epidemiologic variables					
Age in years Median (range)	72 (34‐96)	54 (34‐59)	71 (60‐80)	83 (80‐96)	
Sex (male) N (%)	329 (64%)	36 (63%)	232 (65%)	61 (59%)	.4004
Laboratory variables					
WBC × 10^9^/L Median (range)	11.8 (0.7‐271)	12.8 (2.1‐116.5)	11.3 (0.7‐271)	12.7 (1.6‐156)	.980
Hb g/dL Median (range)	11.0 (4.3‐16.5)	11.1 (6.0‐16.1)	11.1 (4.3‐16.5)	10.5 (5.1‐14.9)	.089
PLT × 10^9^/L Median (range)	108 (2‐1181)	105 (2‐510)	109 (5‐1181)	103 (6‐ 867)	.427
PB Mono % Median (range)	22 (0‐77)	22 (3‐50)	22 (0‐74)	24 (5‐77)	.295
PB Blast % Median (range)	0 (0‐19)	1 (0‐17)	0 (0‐18)	0 (0‐19)	.**008**
Proportion of patients with PB blasts (%)	34%	59.6%	31.8%	29.1%	.**0035**
LDH IU/mL Median (range)	256 (67‐3380)	277 (120‐1952)	255 (68‐3380)	258 (67‐1058)	.275
Biologic variable					
Spont. CFU‐GM/10^5^ PBMNC	6 (0‐1127)	21.5 (0‐762)	5.5 (0‐1127)	7.0 (0‐812)	.931
Mutated genes^a^	Positive cases/total number of cases (proportion in %)				
*NRAS*	34/222 (15%)	1/17 (6%)	26/153 (17%)	7/52 (13%)	.8934
*KRAS*	20/221 (9%)	0/17 (0%)	16/152 (10.5%)	4/52 (7.7%)	.7937
*NF1*	15/196 (7.7%)	1/14 (7.1%)	10/136 (7.4%)	4/46 (8.7%)	.7739
*CBL*	21/222 (9.5%)	2/17 (11.8%)	12/153 (7.8%)	7/52 (13.5%)	.4287
*PTPN11*	12/223 (5.4%)	2/17 (11.8%)	7/153 (4.6%)	3/53 (5.7%)	.6636
*JAK2 V617F*	34/263 (12.9%)	4/22 (18.2%)	28/184 (15.2%)	2/57 (3.5%)	.**0214**
*SETBP1*	47/220 (21.4%)	5/17 (29.4%)	29/151 (19.2%)	13/52 (25%)	.8069
*TET2*	149/221 (67,4%)	9/17 (53%)	104/152 (68.4%)	36/52 (69.2%)	.4014
*DNMT3A*	17/222 (7.7%)	1/17 (5.9%)	9/153 (5.9%)	7/52 13.5%)	.105
*EZH2*	35/221 (15.8%)	1/16 (6.2%)	25/153 (16.3%)	9/52 (17.3%)	.4589
*ASXL1*	54/222 (24.3%)	5/17 (29.4%)	37/153 (24.2%)	12/52 (23.1%)	.6746
*IDH1/2*	11/220 (5%)	1/17 (5.9%)	9/151 (6.0%)	1/52 (1.9%)	.2963
*SRSF2*	43/221 (19.5%)	1/17 (5.9%)	31/152 (20.4%)	11/52 (21.2%)	.3516
*U2AF1*	14/221 (6.3%)	0/17 (0%)	12/153 (7.8%)	2/51 (3.9%)	.8561
*SF3B1*	12/221 (5.4%)	2/17 (11.8%)	8/153 (5.2%)	2/51 (3.9%)	.3296
*ZRSR2*	15/222 (6.8%)	1/17 (5.9%)	11/153 (7.2%)	3/52 (5.8%)	.8375
*RUNX1*	19/222 (8.6%)	1/17 (5.9%)	13/153 (8.5%)	5/52 (9.6%)	.6621
*TP53*	7/204 (3.4%)	0/16 (0%)	5/139 (3.6%)	2/49 (4.1%)	.5637
Cytogenetics					
High risk	52/274 (19%)	12/38 (31.6%)	28/185 (15.1%)	12/51 (23.5%)	.5408

Abbreviations: CFU‐GM, colony‐forming unit‐granulocyte‐macrophage; CMML, chronic myelomonocytic leukaemia; Cytogenetic Risk, high‐risk cytogenetic including trisomy 8, complex karyotype (≥3 abnormalities) and abnormalities of chromosome 7; Hb, haemoglobin; LDH, lactate dehydrogenase; MNC, mononuclear cells; PB, peripheral blood; PLT, platelet count; WBC, white blood cell count.

Significant values are bold.

^a^ At least 5% VAF.

**TABLE 2:** Risk ratios, 95% confidence limits and *P*‐values for univariable competing risk regressions regarding AML transformation

	Risk ratio	Lower 95% confidence limit	Upper 95% confidence limit	*P*‐value
Age categories	0.447	0.313	0.638	<.**001**
Sex	0.898	0.595	1.350	.610
WBC	0.996	0.990	1.000	.240
Hb	0.979	0.896	1.073	.640
PLT	0.997	1.000	1.000	.**029**
Mono	1.010	0.990	1.030	.360
PBblast	1.110	1.062	1.162	<.**001**
LDH	1.000	1.000	1.000	.270
spCFUGM	1.001	1.000	1.000	.360
Cytogenetic risk	1.459	0.827	2.560	.190
AMC	1.000	1.000	1.000	.460
IMC present	1.660	1.073	2.560	.**023**
*ASXL1*	1.478	1.041	2.117	.**031**
Mayo raw score	1.252	1.030	1.522	.**027**

Note

Age categories = age categorised in 3 subgroups, WBC = white blood cell count in G/L, Hb = haemoglobin in g/dL, PLT = platelets in G/L, Mono = peripheral monocyte count, PBblast = presence of peripheral blasts, LDH = lactate dehydrogenase in U/L, spCFUGM = spontaneous growth in colony‐forming units of granulocytic/monocytic precursors, Cytogenetic risk = cytogenetic risk according to Spanish cytogenetic scoring (Such et al), AMC = absolute monocyte count, IMC present = immature monocytes present in peripheral blood, *ASXL1* = presence of nonsense or frameshift *ASXL1* mutation, Mayo raw score = risk according to Mayo prognostic model for CMML without categorisation.

Significant values are bold.

Since for the individualised management of patients with CMML with comorbidities, it is critical to know the patient's actual risk to experience transformation into AML, the possibility of dying without transformation (because of age and comorbidities) was considered as competing risk.

**FIGURE 1:**
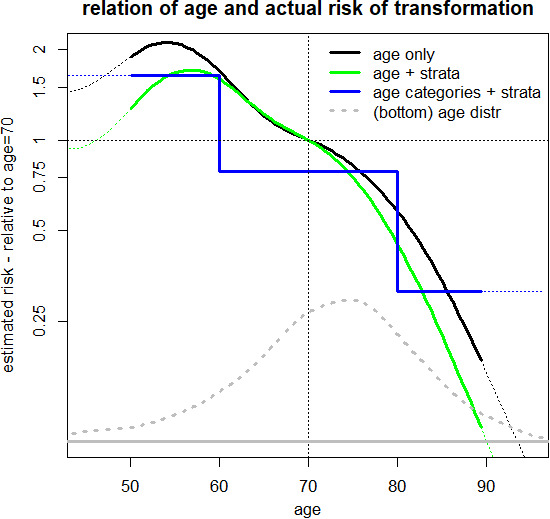
Functional form of the relation between age and risk of transformation. The black curve illustrates the (log)‐linear relation of age to the risk of transformation. The green line shows the risk, adjusted by stratification for the Mayo score, WBC, cytogenetic risk, and *ASXL1* to account for confounding of these variables, the blue line shows the risk for three age categories (stratified as above). The grey dotted line sketches the age distribution of the patients (on linear scales)

For “descriptive purposes”, age was divided into three categories (<60, 60‐79, ≥80 years). These were chosen to best present the estimated relation between age and the actual risk of transformation into sAML (Figure S1). As displayed in Figure 1, age exhibits an essential (log)‐linear relation to the concerned risk, with a risk reduction of roughly 70% between adjacent age categories, after multivariable adjustment for potential confounders, namely the Mayo prognostic model for CMML (abbreviation used: Mayo score, which is based on the parameters haemoglobin <10 g/dL, PLT <100 G/L, absolute monocyte count >10 G/L and circulating immature monocytic cells^22^), WBC, cytogenetic risk, *ASXL1*. There was a significantly declining risk to actually experience transformation with increasing age. To cross‐check the division into three categories, also subdivision into four groups was analysed, but it showed, that there was no meaningful difference in patients aged 60‐69 and 70‐79 (Figure S2). Therefore, we used the 3 age categories <60, 60‐79 and ≥80 years for convenient presentation of results.

**FIGURE 2:**
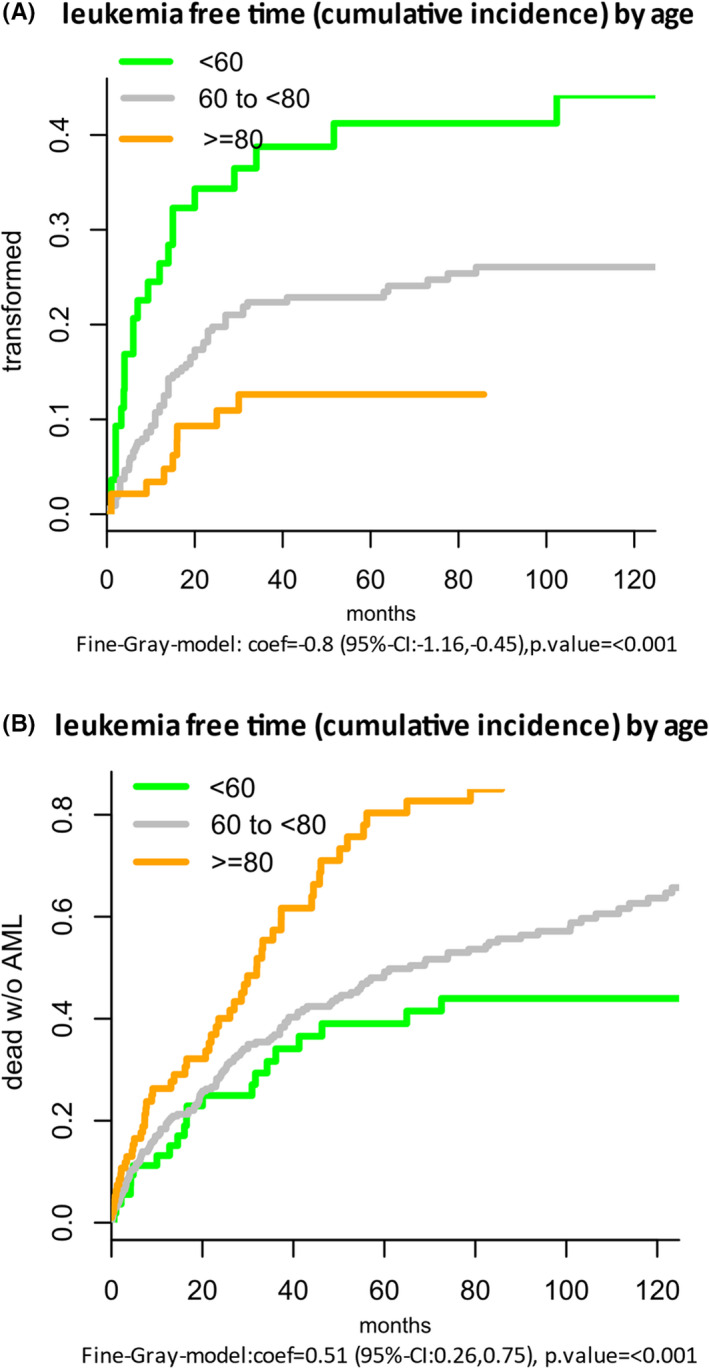
Leukaemia‐free time by age estimated by competing risk analyses. A, Proportion transformed. B, Proportion dead without AML

At 4 years from diagnosis, the estimated proportions of cases transformed into AML were 39% in patients <60 years, 23% in patients 60‐79 and 13% in patients ≥80 years (*P* < .0001). The decrease of the actual risk to experience transformation was partially counterbalanced by a substantial increase in the probability to die without transformation at older age (Figure 2). At 4 years from diagnosis, the estimated proportion of patients who died without AML was 39% in patients <60 years, 43% in patients 60‐79 and 71% in patients ≥80 years (*P* < .0001). The median observation time for the whole population was 53.1 months and 105.7, 53.1 and 34.5 months in the three age groups <60, 60‐79 and ≥80 years, respectively.

Univariable and multivariable competing risk regression models with established single prognostic factors and one established score (Mayo risk score) were calculated. As can be seen in Figure S3 and Table S2, from established prognostic factors including WBC, Hb, PLT, IMC, AMC and high‐risk cytogenetics, only PLT counts, peripheral blasts, IMC, *ASXL1* and the Mayo risk score were significantly predictive regarding the cumulative risk of transformation.

In order to substantiate potential age effects, also the correlation of each prognostic factor with age was estimated by Kendall's tau (see Table S2). Most of these showed at most slightly lower values at older age, significant only regarding Hb, peripheral blasts and *ASXL1*.

Models for the actual risk to experience transformation considering age with one established prognostic factor at a time showed that higher age maintains a substantial and significant lowering impact on risk also in combination with each of the considered prognostic indicators (see Table S3), regardless of being categorised or not, while only platelets, peripheral blasts, *ASXL1* and the Mayo score reached significance in combination with age. In the analysed combinations, the risk to transform to sAML was reduced by roughly 30% per ten additional years of age.

To make the previously described results clinically applicable, they were combined in a score for actual risk of transformation (ART score) based on a multivariable Fine and Gray model with age, peripheral blasts and platelets as risk indicators. *ASXL1* mutation was considered as potential predictor, but it did not enhance the identification of patient subgroups with minimal actual transformation risk and was therefore not included in the score. For descriptive purposes and clinical convenience, the ART score was divided into three categories (low, intermediate and high) see Table 3.

**TABLE 3:** Formula for the ART Score (actual risk of transformation score). Based on the cut points given, the score divides CMML patients in three subgroups (low, intermediate and high)

Formula: ART score = 18 − age/5 + PB blast ‐ PLT/100	
Age = age in years, PB blast = peripheral blasts in per cent, PLT = platelets in G/L	
Category	Cut points
Low	≤0
Int	>0 to ≤3
High	>3

Note

See Table S4, for example, calculations of the ART score.

Abbreviations: Age, age in years; PB blast, peripheral blasts in per cent; PLT, platelets in G/L.

**FIGURE 3:**
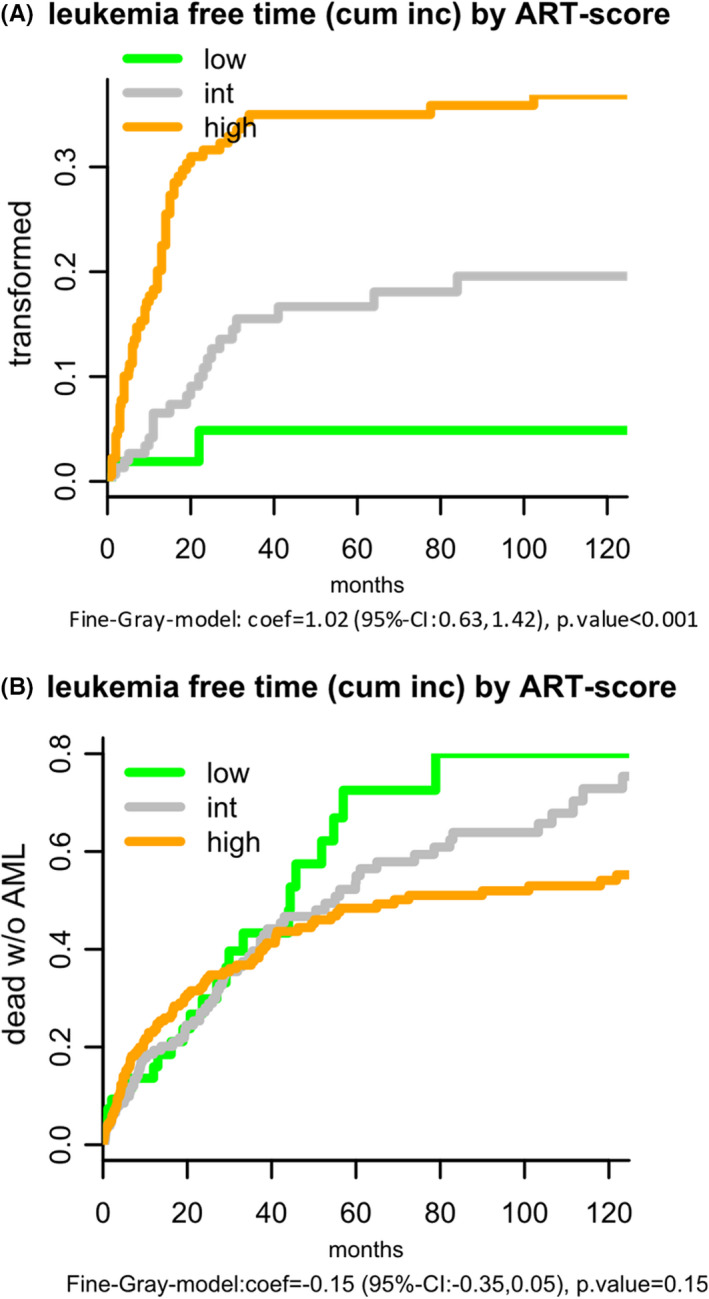
A, B, Leukaemia‐free time by ART score depending on three risk categories. A, Proportion transformed. B, Proportion dead without AML

Figure 3A and the Table S5 show the estimated risk of AML transformation over time depending on the risk categories. Figure 3B and Table S6 show the estimated proportions of deaths without AML transformation. The opposite trend between the AML risk over time and the risk of dying without AML is illustrated. In comparison, Figure S3A,B show the estimated risk of AML transformation over time predicted by the Mayo Score.

The ART score including age as one component is (necessarily but only) moderately correlated with age (tau = −0.45) (see Table S7 and Figure S4A,B). In the age group ≥80, about 40% are assigned to low risk, while in the age group <60 no one is categorised into "low," but 94% into "high." Example calculations using the ART score are shown in Table S4.

### 3.1 Proving of the stability by the Bootstrap method

As internal validation of the proposed ART score, several steps in its development were checked by bootstrapping. This was done by creating 1000 bootstrap samples of the original data by randomly including cases from the database and repeating the modelling steps in each of the 1000 samples. The estimated weights for the score components, and more importantly, the estimated transformation probabilities for each risk category at different time points were quite stable (see Tables S8 and S9). For example, the 95%‐bootstrap‐confidence intervals for the transformation probability at 48 months by ART score risk categories were for low risk: 0‐0.13, intermediate: 0.10‐0.24, high: 0.27‐0.42.

## 4 DISCUSSION

The management of cancer in older aged people is becoming a common challenge due to the ageing of the population.^23^ There are many interconnected variables that may contribute to the complex situation in older patients suffering from haematological malignancies. CMML is a typical example for a malignancy, in which the risk of dying from progression of blood cancer (sAML) has to be viewed in relation to the risk of mortality unrelated to sAML. The primary aim of CMML treatment with HMA is to delay transformation into sAML. Since HMA treatment may be associated with significant inconvenience, particularly for elderly patients, it is clinically highly desirable to know if and when the patient will experience this complication in the future.

The data of the ABCMML registry match with the results of an international CMML database merging CMML registries from 8 tertiary care centres of three different countries (USA, France, Italy) with data on 1832 patients. The median age at diagnosis of these patients was 70 (16‐93 years) with a male predominance (67%), the median OS of the entire data set was 31 months, and leukaemia transformations were observed in 21% of the patients at last follow‐up. Comparing different CMML prognostic scoring systems, no statistical difference in those models containing *ASXL1* mutation was identified by the authors in this large cohort compared with models containing clinical variables alone.^1^

The actual risk to experience transformation to sAML depends on both, the transformative aggressiveness of the disease and on the competing risk of dying before transformation, mainly by comorbidities and sometimes also by the consequences of severe CMML‐related cytopenias. By competing risk regression as introduced by Fine and Gray, we analysed the impact of age and further features on the actual risk of transformation.

Age was found to have a major impact on the actual risk (also called cumulative incidence) of transformation of CMML to sAML, that is with rising age, the actual risk to transform declines. This seems to be partly, but not solely, due to the increased mortality by the comorbidities. Splitting patients into three age categories showed a significantly declining transformation risk with higher age. In our sample, CMML patients 80 years or older have a cumulative transformation incidence at four years of only 13%. The favourable effect of older age persists, even if other clinical or molecular variables are taken into account and combining age with other features was expected to offer an even better discrimination of patients with low actual transformation risk.

Several prognostic parameters and risk scores have been reported for patients with CMML.^22^, ^24^, ^25^, ^26^, ^27^, ^28^, ^29^, ^30^, ^31^ Their main focus was to predict survival, time to transformation and to a lesser extent leukaemia‐free survival. This is reasonable, as the main focus of these papers was to identify high‐risk patients, who would require intensive therapy, assuming that they will be alive long enough to be at substantial risk of transformation. Although not explicitly stated, one could say that they aimed at estimating the severity of the disease. In this sense, time to transformation as well as overall survival, and leukaemia‐free survival can be understood as surrogate markers for the clinical impact of the disease.

Consequently, only few of these papers applied also competing risk models and no main result was based on these techniques. A typical CMML risk score designed to identify patients who qualify for disease‐modifying therapy would therefore for good reasons not include age, as it would increase the risk estimate in older age, especially in models for overall survival, which if applied in risk‐based treatment decisions is inadequate.^32^

Our interest, in contrast, was primarily to detect those patients, who, because of old age and other readily available clinical aspects, show a low probability to experience transformation into sAML. Therefore, we intended to enhance the prognostic power of age by combining it with other features into a simple practical score.

In our analysis, the platelet count and peripheral blasts were found to further improve prediction additionally to age. Also, not unexpectedly, the presence of a molecular aberration of the *ASXL1* gene was predictive, but it would mainly enhance the discrimination between intermediate and high transformation risk. Therefore, *ASXL1* was not included in our proposed score. Other molecular parameters did not show an adverse impact. Thus, our analysis resulted in a simple non‐invasive score that can be easily applied and communicated to the patient as soon as the PB counts and the differential count are available.

It seems useful to identify a group of CMML patients with a very low risk to experience transformation to sAML. We show that CMML patients 80 years or older with a normal platelet count and no blast cells in PB have only a minimal risk of dying from sAML. This group of patients comprises around 15% of all CMML patients, and these patients are most likely candidates in whom a watch‐and‐wait strategy may sometimes be reasonable or even preferable over cytoreductive therapies that may even be avoided by careful monitoring the course in these patients. Interestingly, the Mayo Score, one of the most established prognostic scores in patients with CMML, due to its different aim, was less effective in identifying a patient subset with a minimal actual transformation risk.

The lower risk of older patients to actually experience a transformation may be due to a higher non‐transformation‐related mortality in this subgroup. However, it may also indicate a lower aggressiveness of the disease in older patients, which, based on our results, cannot be precluded either. In this context, the survival of patients <60 years was unexpectedly low compared to published data on “younger” CMML patients <65 years.^34^ This may be partially due to study sample differences regarding risk factors, the decades covered by our data, and related to this, available therapeutic options. In the collaborative study of Patnaik et al, the median overall survival for the whole CMML population <65 years was 55 months, whereas the subgroup of patients with CMML 2 had a poorer OS of 24 months. It seems that younger patients included in our database had more advanced disease, with two thirds of them presenting with blast cells in the peripheral blood. There are reports in the literature which may be in agreement with this observation in other haematological malignancies. Thus, older patients with AML present with lower WBC and a lower percentage of marrow blasts.^33^ Moreover, from informative patient records we saw that around half of the CMML patients younger than 60 years received intensive chemotherapy and part of them underwent stem cell transplantation. Results from an international cohort including 1653 CMML patients which were recently published showed that patients treated with intensive chemotherapy had a very poor survival with a median OS of only 11 months.^12^ Furthermore, we cannot exclude a selection bias favouring referral of younger patients with more clinically aggressive disease by physicians in the community.

In order to arrive at a stable estimate regarding the actual transformation risk, we used peripheral blasts instead of bone marrow blasts for several reasons. The determination of peripheral blasts, not requiring a bone marrow puncture, allows repeated application of the ART score. The determination of the sum of blasts and promonocytes seems to be more reliable in PB than in BM, when different pathologists are involved in the evaluation of CMML patients,^35^ which was the case in our study and would necessarily be in general clinical application of the score. Also in the multivariable analysis reported by the Mayo group ^22^ for their prognostic model, PB blasts but not BM blasts had a significant impact on leukaemia‐free survival.

One limitation, however, was, that because of incomplete data on the exact blast cell content in bone marrow we could not conclusively check in the present study, if the bone marrow blast percentage would add prognostic power to the suggested score.

Furthermore, the proposed ART score was developed in an exploratory analysis on retrospective data. Although all crucial steps of the development were internally validated by bootstrapping, a validation study by an independent study group would be highly appreciated and required to confirm our observation. In a real‐life collection such as the ABCMML, not all data which would be obtained in a systematic prospective study are available. However, real‐life data have been recently recognised as an important way to get insights into the natural history of rare diseases.^36^ On the other hand, the ABCMML provides information derived from molecular and from functional studies and therefore allows a more comprehensive view and deeper insight into the complex pathophysiology of this haematological malignancy.^13^

## CONFLICT OF INTEREST

The authors declare that they have no conflict of interest to disclose in this study.

## AUTHOR CONTRIBUTION

SMS, KG and HT wrote the manuscript; EJ performed colony assays; AB, MG and RM performed NGS analyses; TG and EG performed the administration of data; GU, OZ and GW interpreted molecular data; CG, TN, MP, PB, AW, SH, JT, AZ, HS, RS, RK, EU, BS, LÖ, UG and PV provided patient samples and clinical information; HT performed the statistical analyses and developed the ART score; KG directed the research, collected, analysed and interpreted the data. All authors have read and agreed to the published version of the manuscript.

## Supporting information

Supplementary Material

## Data Availability

The data that support the findings of this study are available from the corresponding author, [KG], upon reasonable request.
