# Supplementary material for: Impact of age on the cumulative risk of transformation in patients with chronic myelomonocytic leukaemia
Source: Eur J Haematol. 2021 Jun 1;107(2):265–74. doi: 10.1111/ejh.13647 (PMC8480146; doi:10.1111/ejh.13647)
Supplement: Supplementary file 1 — Supplementary Material [file EJH-107-265-s001.docx]

**Supplemental information**

Supplemental Figure 1A.

Kaplan Meier estimates of time to transformation into secondary AML in CMML patients stratified by age (division into 3 age categories < 60, 60-79, >= 80 years)


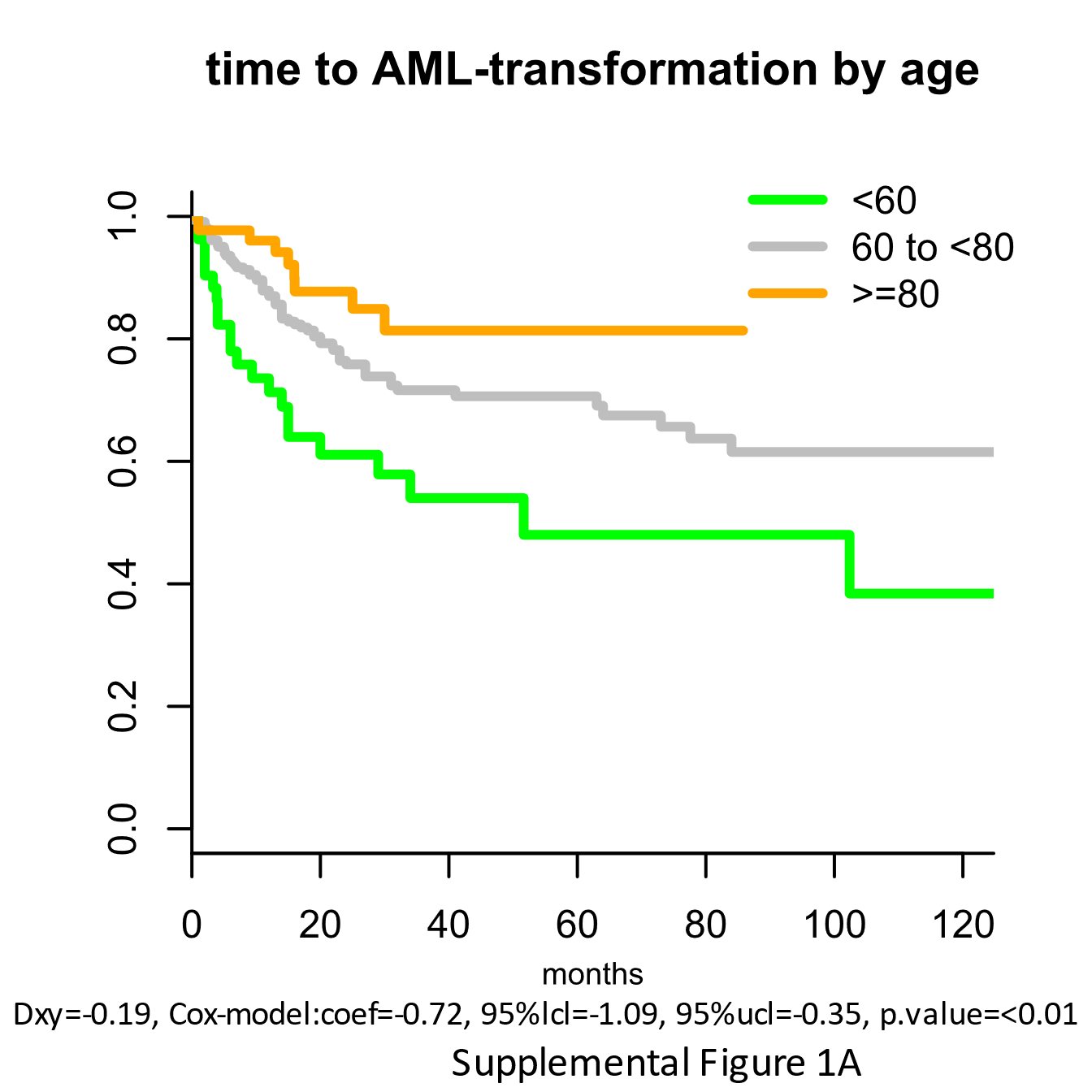


Supplemental Figure 1B. Kaplan Meier estimates of overall survival in CMML patients stratified by age (division into 3 age categories < 60, 60-79, >= 80 years)


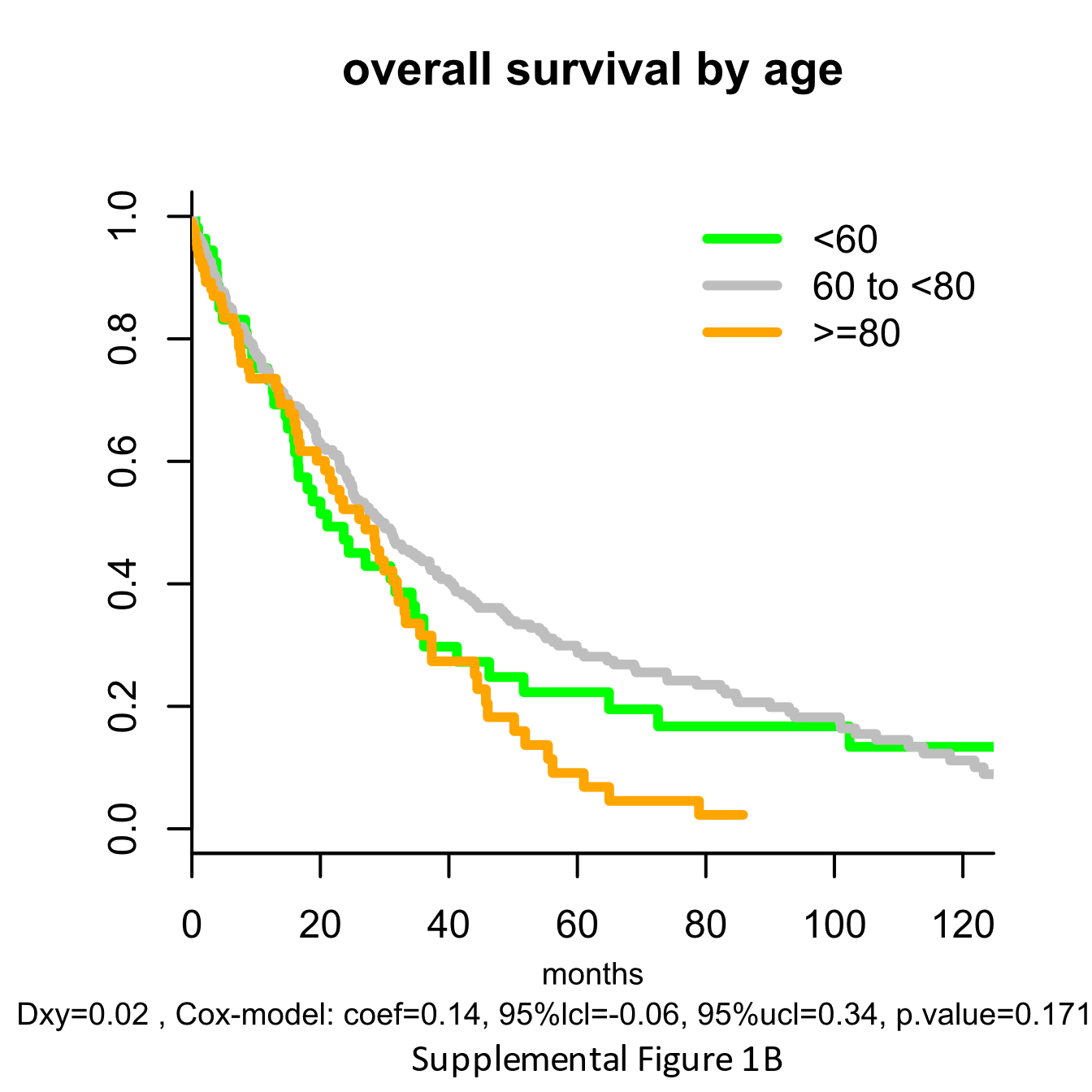


The supplemental figure 1A shows hypothetical time to transformation curves, based on the cause-specific hazard, and in the bottom line the coefficient for a respective Cox-model is given (0.72). Since exp(0.72) = 0.49, it means that the hazard of transformation for a higher age category is about only half of that for the next lower age category. In comparison, the Cox coefficient for the Mayo-score would be 0.74, which means that the hazard for the next higher Mayo-category is about twice as high as for the next lower category, as exp(.74)=2.1 . Taking the different proportions of the Mayo-categories within the age groups into account, the coefficient for age does not change substantially - the estimated proportion changes from 0.49 to 0.52 .
In combination with the supplemental figure 1B, the results indicate that the difference in the actual probability for a transformation is mainly due to the substantially lower hazard of transformation in higher age groups.

Supplemental Figure 2: Cumulative transformation risk calculated by competing risk analysis in CMML patients stratified into 4 age categories (<60 years, 60 to <70, 70 to <80 and >= 80 years)


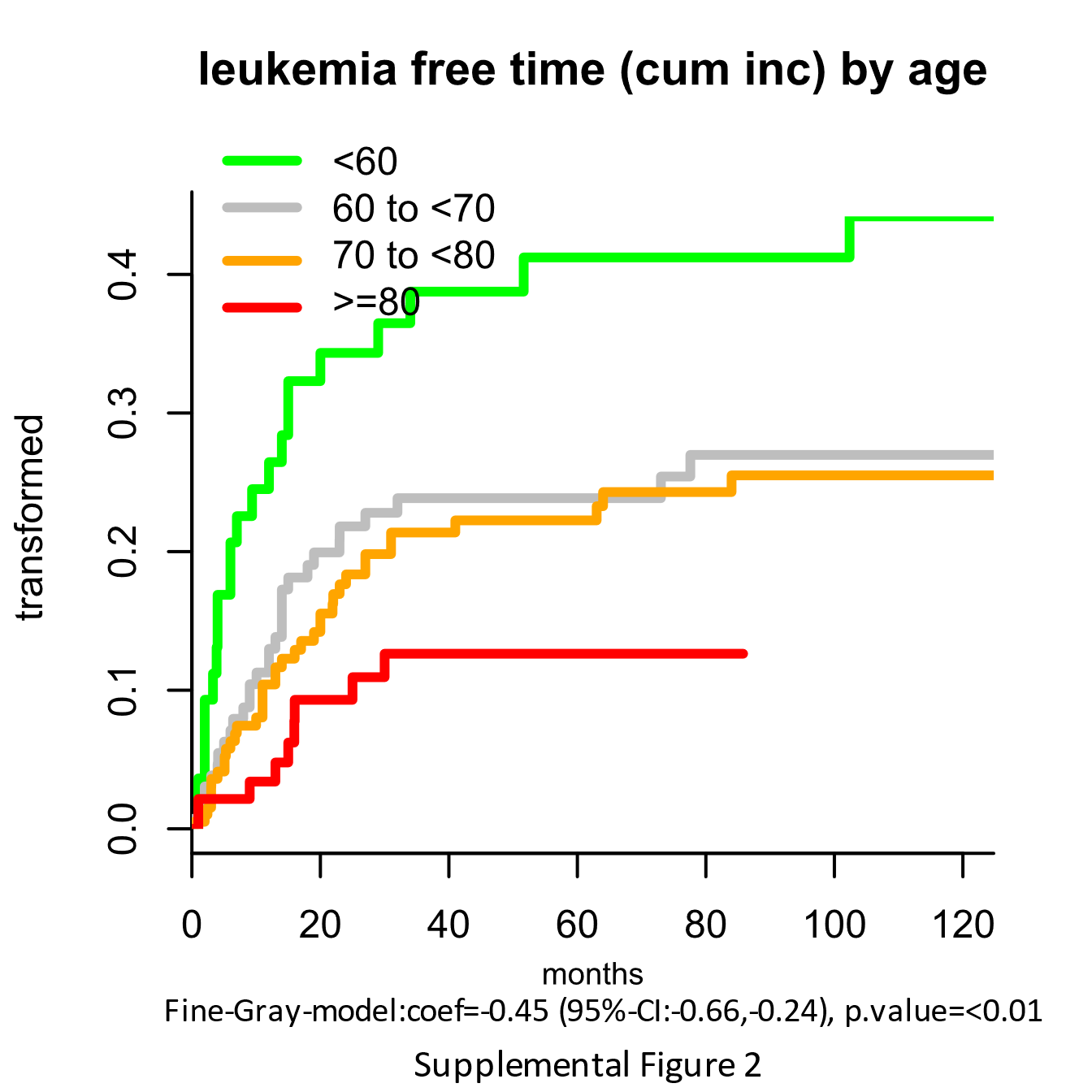


Supplemental figure 3: Cumulative risk of transformation calculated by competing risk analysis in CMML patients stratified by Mayo risk score (low, intermediate, high risk)

Supplemental Figure 3A: proportion transformed


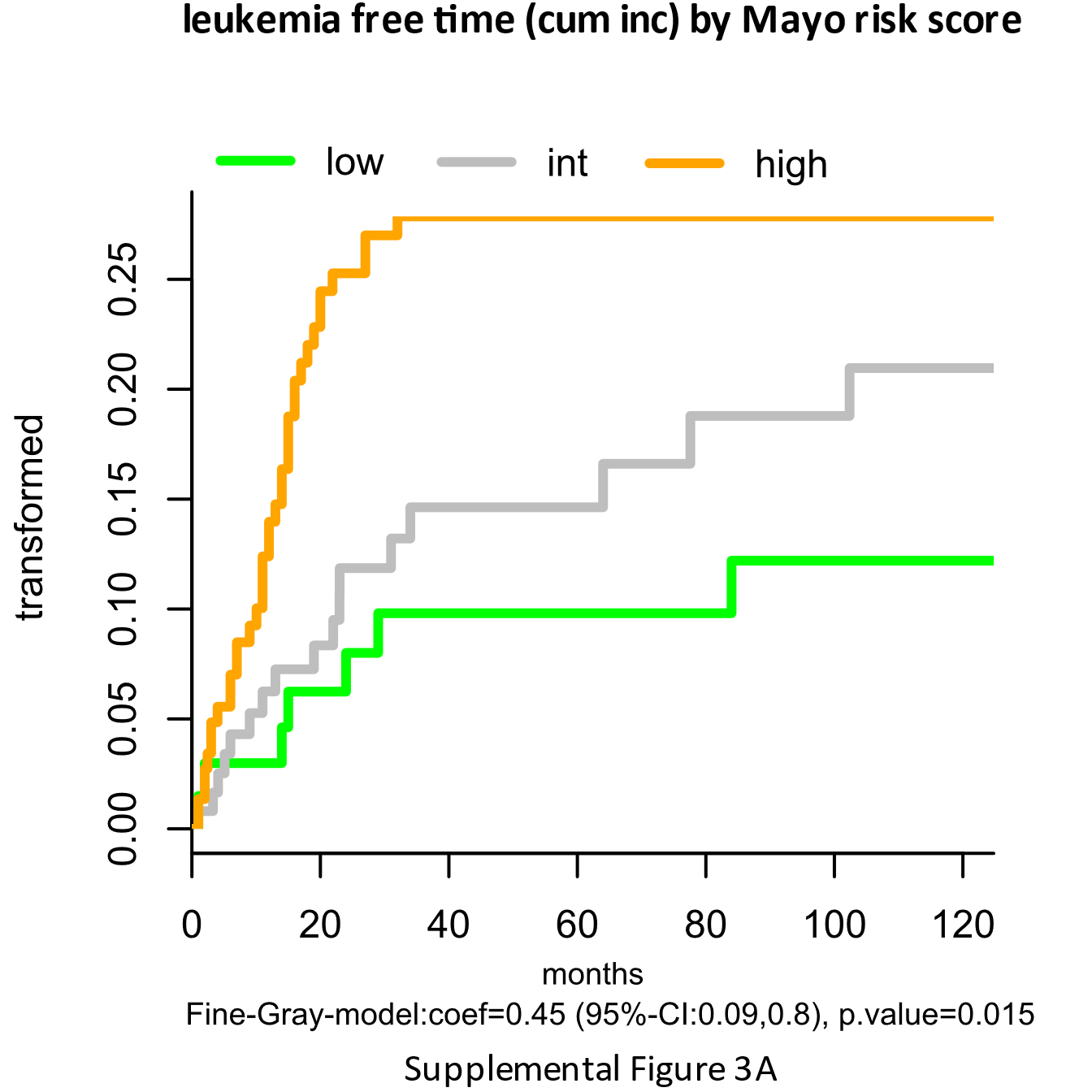


Supplemental Figure 3B: proportion dead without AML


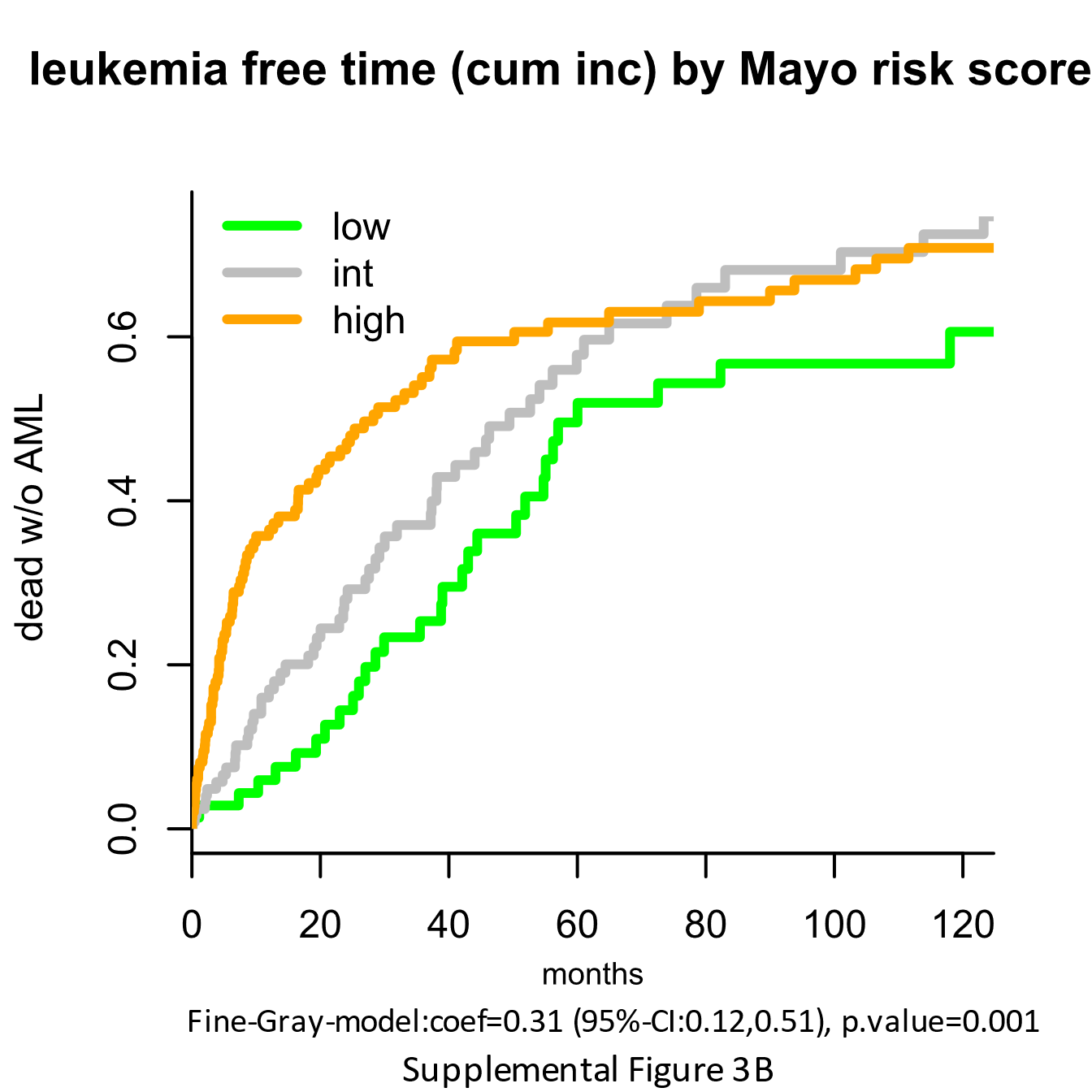


Supplemental figures 4A and 4B:

Distribution of the patients into the 3 risk categories of the ART score (low, intermediate, high) in the age groups <60 years, 60-79 years and >= 80 years

Supplemental Figure 4A:


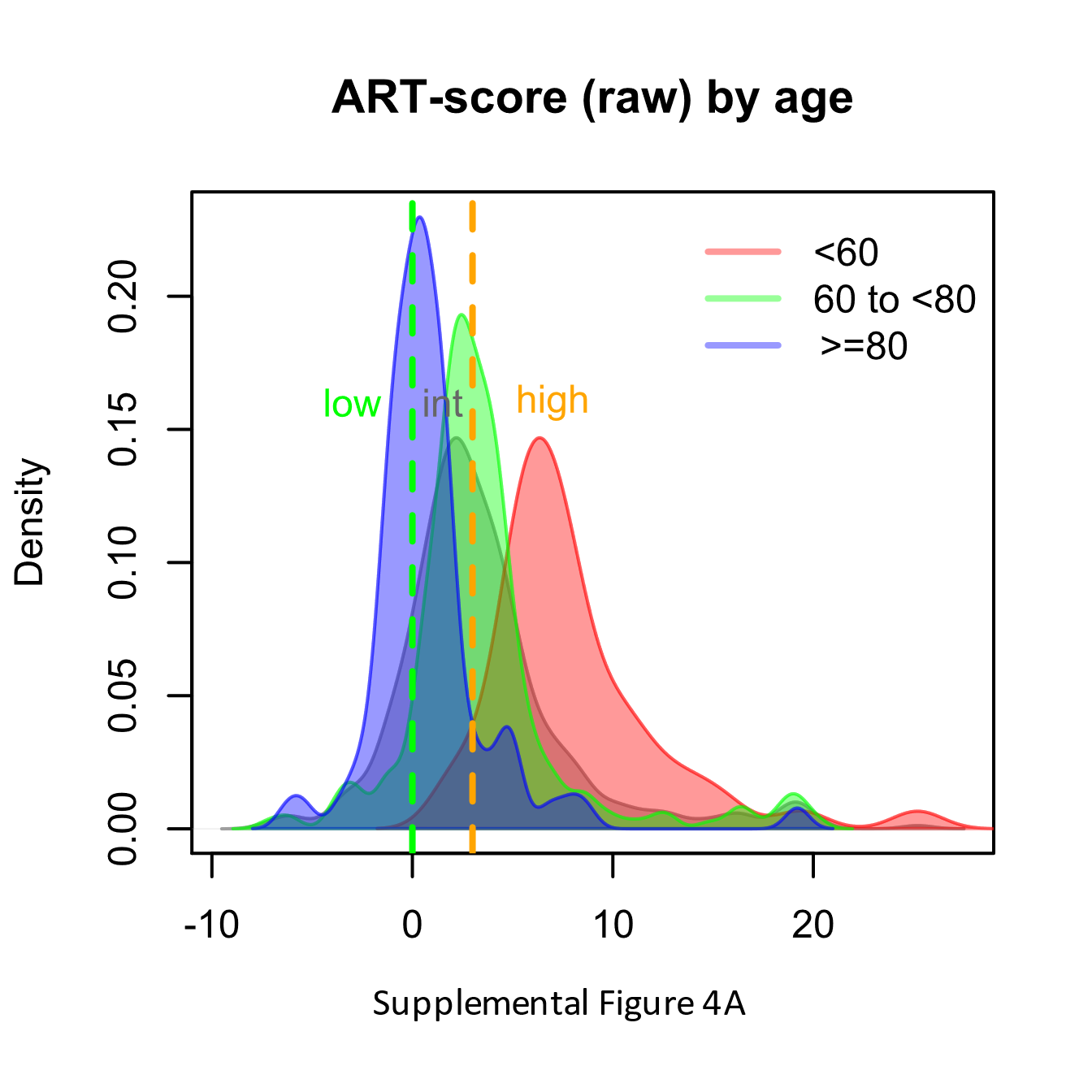


Supplemental Figure 4B


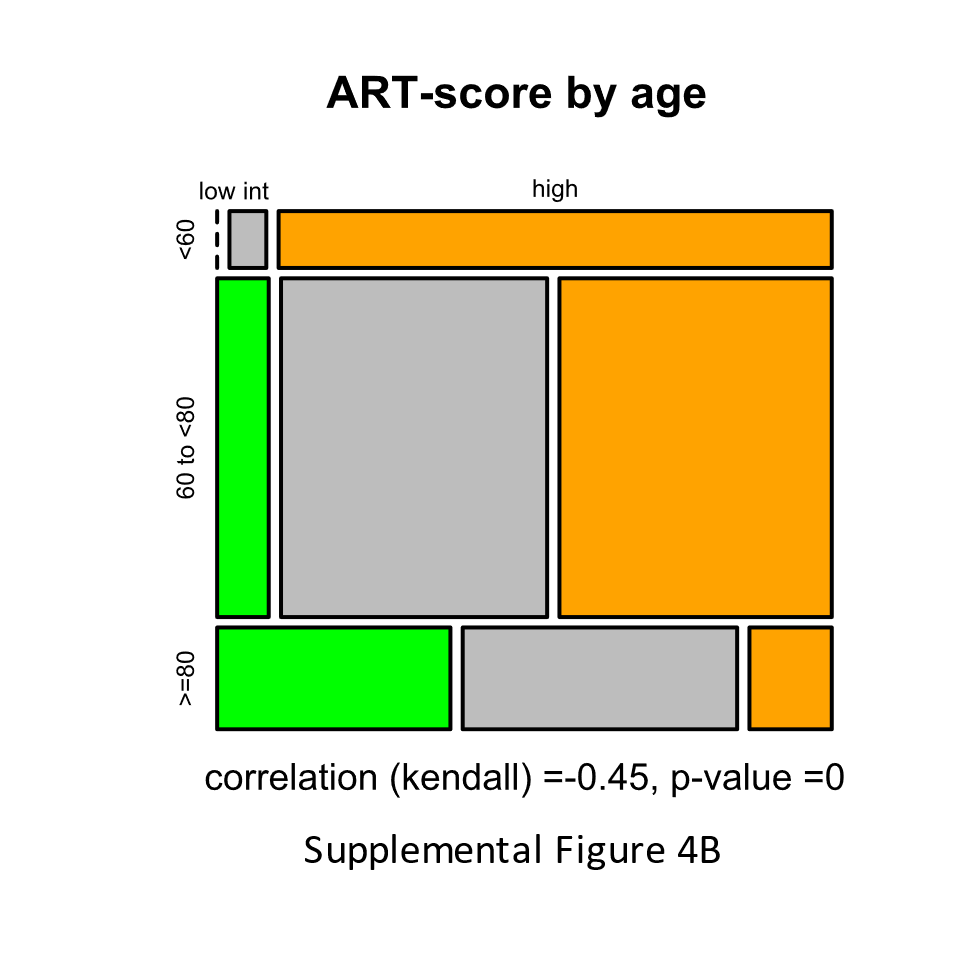


Supplemental figure 5:

Distribution of the patients into the 3 risk categories of the Mayo risk score by the ART-score (X-axis: ART score, Y-axis: Mayo risk score)


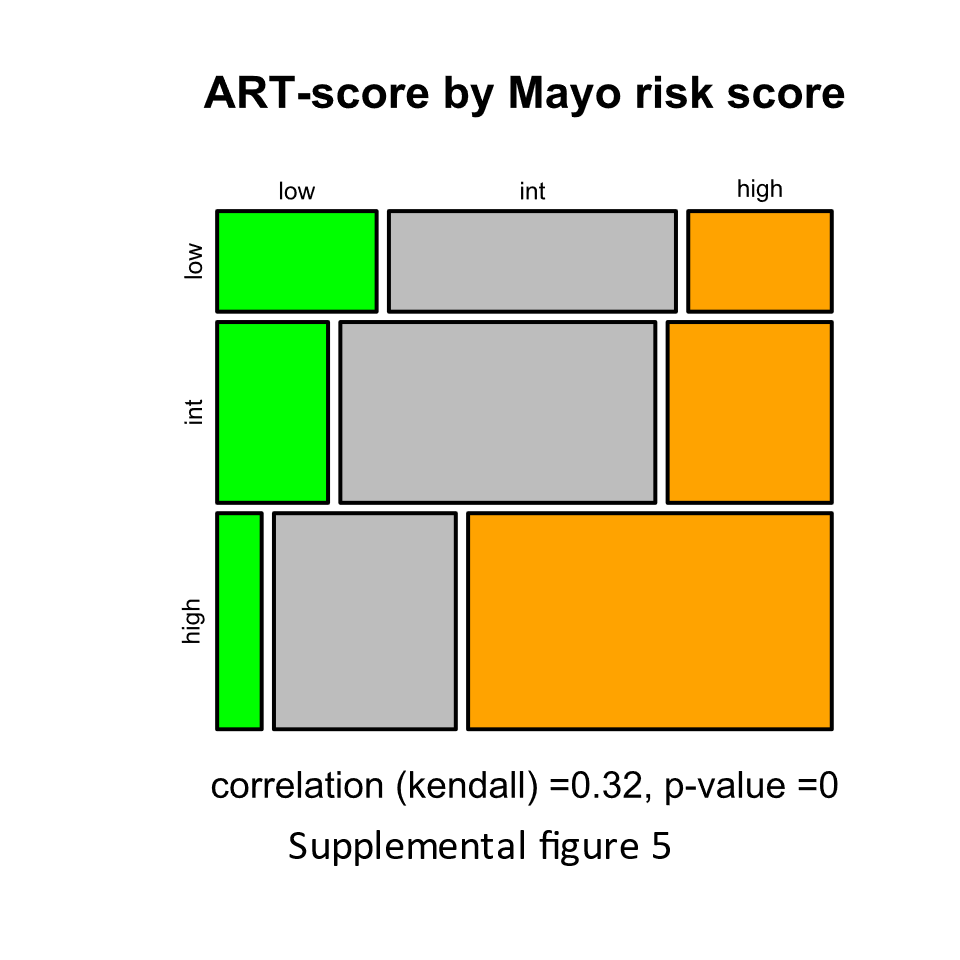


## Supplemental Table 1A: absolute number of patients with peripheral blasts in the 3 age categories (<60, 60-79 and >=80 years)

| age categories (years) | no blasts in PB | blasts in PB | total number |
| --- | --- | --- | --- |
| <60 | 19 | 28 | 47 |
| 60 to 79 | 193 | 90 | 283 |
| >=80 | 61 | 25 | 86 |
| total | 273 | 143 | 416 |

## Supplemental Table 1B: peripheral blast count in percent in the 3 age categories <60 years , 60-79 years and >=80

| age categories (years) | no blasts in PB (%) | blasts in PB (%) | proportion of patients |
| --- | --- | --- | --- |
| <60 | 40.4% | 59.6% | 11.3% |
| 60 to 79 | 68.2% | 31.8% | 68.0% |
| >=80 | 70.9% | 29.1% | 20.7% |
| Total (mean) | 65.6% | 34.4% | 100,0% |

correlation ( kendall ) = -0.14 , p-value = 0.0035

Cramer's V =0.12 p-value(chi-square-test)=5e-04

Supplemental table 2: Kendall's tau correlations for prognostic factors with age

|  | tau | p-value |
| --- | --- | --- |
| Sex | 0.053 | 0.142 |
| WBC | 0.005 | 0.877 |
| Hb | -0.064 | **0.035** |
| PLT | -0.024 | 0.431 |
| Monocyte count | 0.043 | 0.179 |
| PBblast (IMC) | -0.102 | **0.007** |
| LDH | -0.047 | 0.205 |
| spCFUGM | 0.001 | 0.980 |
| Cytogenetic risk | -0.065 | 0.189 |
| AMC | 0.036 | 0.258 |
| IMC present | -0.066 | 0.100 |
| *ASXL1* | -0.106 | **0.050** |
| Mayo raw score | 0.001 | 0.973 |

Legend Supplemental Table 2: WBC= white blood cell count in G/L, Hb= hemoglobin in g/dL, PLT= platelets in G/L, Monocyte count= peripheral monocyte count, PBblast (IMC)= presence of peripheral blasts/ immature monocytic cells, LDH= lactate dehydrogenase in U/L, spCFUGM= spontaneous growth in colony forming units of granulocytic/ monocytic precursors, Cytogenetic risk= cytogenetic risk according to Spanish cytogenetic scoring (Such et al), AMC= absolute monocyte count in G/L, IMC present= immature monocytes present in peripheral blood, *ASXL1*= presence of *ASXL1* nonsense or frameshift mutation, Mayo raw score= risk according to Mayo prognostic model for CMML without categorization

Supplemental table 3: risk ratios and p-values for each variable for bivariable competing risk regressions with age as additional variable

|  | Risk ratio (for variable in first column) | Age (3 groups) | p-value for risk ratio of variable in first column | p-value for age |
| --- | --- | --- | --- | --- |
| Sex | 0.925 | 0.448 | 0.710 | <0.001 |
| WBC | 0.996 | 0.465 | 0.270 | <0.001 |
| Hb | 0.973 | 0.475 | 0.550 | <0.001 |
| PLT | 0.997 | 0.451 | 0.026 | <0.001 |
| Mono | 1.010 | 0.400 | 0.330 | <0.001 |
| PBblast | 1.110 | 0.474 | 0.000 | <0.001 |
| LDH | 1.000 | 0.454 | 0.440 | <0.001 |
| spCFUGM | 1.001 | 0.332 | 0.540 | <0.001 |
| Cytogenetic risk | 1.415 | 0.571 | 0.230 | 0.007 |
| AMC | 1.000 | 0.395 | 0.610 | <0.001 |
| IMC present | 1.525 | 0.513 | 0.057 | <0.001 |
| *ASXL1* | 1.456 | 0.337 | 0.032 | <0.001 |
| Mayo raw score | 1.225 | 0.415 | 0.046 | <0.001 |

Legend Supplemental Table 3: Age categories = age categorized in 3 subgroups, WBC= white blood cell count in G/L, Hb= hemoglobin in g/dL, PLT= platelets in G/L, Mono= peripheral monocyte count in G/L, PBblast= presence of peripheral blasts, LDH= lactate dehydrogenase in U/l, spCFUGM= spontaneous growth in colony forming units of granulocytic/ monocytic precursors, Cytogenetic risk= cytogenetic risk according to Spanish cytogenetic scoring (Such et al), AMC= absolute monocyte count, IMC present= immature monocytes present in peripheral blood, *ASXL1*= presence of *ASXL1* nonsense or frameshift mutation, Mayo raw score= risk according to Mayo prognostic model for CMML without categorization

Supplemental table 4:

Example calculations of the ART-score

Formula: ART-score = 18 - age/5 + PBblast - PLT/100

| Parameters | Age in years | PB blasts in percent | PLT in G/L | ART score | ART category |
| --- | --- | --- | --- | --- | --- |
| Example 1 | 85 | 0 | 100 | 0 | low |
| Example 2 | 80 | 0 | 300 | -1 | low |
| Example 3 | 75 | 0 | 300 | 0 | low |
| Example 4 | 65 | 1 | 300 | 3.0 | intermediate |
| Example 5 | 50 | 2 | 100 | 10.0 | high |

## Supplemental table 5

## leukemia free time (competing risk) by ART-score - see figure 3A

event: transformed

| category | number | number of events | rmean |
| --- | --- | --- | --- |
| low | 59 | 2 | 10.223 |
| intermediate | 172 | 22 | 38.594 |
| high | 189 | 63 | 79.736 |

note: rmean = mean time in state, restricted (max time =224)

event: probability for transformation depending on time

| ART score | 12 months | 24 months | 48 months | 96 months |
| --- | --- | --- | --- | --- |
| low | 0.02 | 0.05 | 0.05 | 0.05 |
| intermediate | 0.07 | 0.12 | 0.17 | 0.20 |
| high | 0.20 | 0.32 | 0.35 | 0.36 |

Fine-Gray: coef=1.02 (95%-CI:0.63,1.42), p.value=0 , valid n: 420

Gray-test (p-value)=0, (event: transformed)

## Supplemental table 6:

## leukemia free time (competing risk) by ART-score- see figure 3B

event: dead without AML

| risk category | number | n event | r mean |
| --- | --- | --- | --- |
| low | 59 | 24 | 151.59 |
| intermediate | 172 | 75 | 138.19 |
| high | 189 | 85 | 109.45 |

note: rmean = mean time in state, restricted (max time =224)

event: probability for dead without AML by ART score (depending on time)

| risk category | 12 months | 24 months | 48 months | 96 months |
| --- | --- | --- | --- | --- |
| low | 0.16 | 0.30 | 0.57 | 0.80 |
| intermediate | 0.19 | 0.27 | 0.47 | 0.64 |
| high | 0.23 | 0.33 | 0.44 | 0.52 |

Fine-Gray: coef=-0.15 (95%-CI:-0.35,0.05), p.value=0.15 , valid n: 420

Gray-test (p-value)=0.224, (event: dead w/o AML)

Supplemental table 7:

Absolute number of patients in the 3 categories according to ART score:

| age categories | low | intermediate | high | total |
| --- | --- | --- | --- | --- |
| <60 | 0 | 3 | 45 | 48 |
| 60 to <80 | 25 | 129 | 132 | 286 |
| >=80 | 34 | 40 | 12 | 86 |
| total | 59 | 172 | 189 | 420 |

Proportion of patients (in percent) in the 3 categories according to ART score

| age categories | low | intermediate | high | total |
| --- | --- | --- | --- | --- |
| <60 | 0,0 | 6,2 | 93,8 | 11,4 |
| 60 to <80 | 8,7 | 45,1 | 46,2 | 68,1 |
| >=80 | 39,5 | 46,5 | 14,0 | 20,5 |
| total | 14,0 | 41,0 | 45,0 | 100,0 |

correlation (kendall) = -0.45 , p-value = 0

Supplemental Table 8

Bootstrap results:
comparison of original- and bootstrap estimates for the weights of each score component and the categorised score, as well as the Dxy for the models

| variable | original | boot | bias=b-o | sd.boot |
| --- | --- | --- | --- | --- |
| age | -0.0369549 | -0.0374850 | -0.00053004 | 0.0098850 |
| PBblast | 0.1024175 | 0.1037986 | 0.00138112 | 0.0244878 |
| PLT | -0.0022859 | -0.0024482 | -0.00016227 | 0.0012128 |
| Dxy | 0.3916584 | 0.3930522 | 0.00139375 | 0.0571546 |
| ART-score | 1.03178 | 1.03891 | 0.0071329 | 0.202842 |
| Dxy | 0.32103 | 0.31899 | -0.0020473 | 0.047058 |

Legend: The column "original" contains the estimates of the original model. The column "boot" shows the mean estimates from bootstrap-models. "bias=b-o" describes the difference between the original estimates and the mean bootstrap estimates. "sd.boot" gives the standard deviation of the bootstrap estimates to guide the interpretation of the bias. Since in each variable the absolute bias is much smaller than the sd.boot, the biases can be regarded as negligible

Supplemental Table 9
Bootstrap: cumulative incidence - bootstrap results with 95% confidence intervals - separately by each risk category

| time (months) | 12 | 24 | 48 | 96 |
| --- | --- | --- | --- | --- |
| low risk category |  |  |  |  |
| original | 0.02 | 0.05 | 0.05 | 0.05 |
| boot | 0.02 | 0.05 | 0.05 | 0.03 |
| lcl (95%) | 0.00 | 0.00 | 0.00 | 0.00 |
| ucl (95%) | 0.06 | 0.13 | 0.13 | 0.11 |
| intermediate risk category |  |  |  |  |
| original | 0.07 | 0.12 | 0.17 | 0.20 |
| boot | 0.07 | 0.12 | 0.17 | 0.20 |
| lcl (95%) | 0.03 | 0.06 | 0.10 | 0.12 |
| ucl (95%) | 0.11 | 0.18 | 0.24 | 0.28 |
| high risk category |  |  |  |  |
| original | 0.20 | 0.32 | 0.35 | 0.36 |
| boot | 0.20 | 0.32 | 0.35 | 0.36 |
| lcl (95%) | 0.14 | 0.24 | 0.27 | 0.28 |
| ucl (95%) | 0.26 | 0.38 | 0.42 | 0.43 |

Legend Supplemental Table 9:

The line "original" contains the estimates of the original model. The line "boot" shows the mean estimates from bootstrap-models. Lcl= lower confidence limit, ucl= upper confidence limit
